# ^18^F-FDG-PET in Mouse Models of Alzheimer's Disease

**DOI:** 10.3389/fmed.2019.00071

**Published:** 2019-04-18

**Authors:** Caroline Bouter, Yvonne Bouter

**Affiliations:** ^1^Department of Nuclear Medicine, University Medical Center Göttingen, Georg-August-University, Göttingen, Germany; ^2^Division of Molecular Psychiatry, Department of Psychiatry and Psychotherapy, University Medical Center Göttingen, Georg-August-University, Göttingen, Germany

**Keywords:** ^18^F-FDG-PET, Alzheimer's disease, mouse model, PET, APP, presenilin

## Abstract

Suitable animal models and *in vivo* biomarkers are essential for development and evaluation of new therapeutic strategies in Alzheimer's disease (AD). ^18^F-Fluorodeoxyglucose (^18^F-FDG)-positron-emission tomography (PET) is an imaging biomarker that allows the assessment of cerebral glucose metabolism *in vivo*. While ^18^F-FDG-PET/CT is an established tool in the evaluation of AD patients, its role in preclinical studies with AD mouse models remains unclear. Here, we want to review available studies on ^18^F-FDG-PET/CT in AD mouse models in order to evaluate the method and its impact in preclinical AD research. Only a limited number of studies using ^18^F-FDG-PET in AD mice were carried out so far showing contradictory findings in cerebral FDG uptake. Methodological differences as well as underlying pathological features of used mouse models seem to be accountable for those varying results. However, ^18^F-FDG-PET can be a valuable tool in longitudinal *in vivo* therapy monitoring with a lot of potential for future studies.

## Introduction

Assessment of neuronal dysfunction with the glucose analog ^18^F-Fluorodeoxyglucose (^18^F-FDG) is a well-established imaging method in the differential diagnosis of neurodegenerative diseases. ^18^F-FDG-PET displays the rate of cerebral glucose metabolism which is mainly determined by synaptic activity (1, 2). Impairment of synaptic function and neuron loss are major pathological hallmarks of AD and other dementias. ^18^F-FDG-PET can be used to identify specific patterns of neuronal dysfunction characteristic for AD with high sensitivity and specificity due to reduced glucose metabolism in certain cerebral areas. Patterns of decreased glucose metabolism mainly include parietal, temporal and frontal cortical areas (3).

Using small animal PET scanners, the same molecular processes can be measured in animal models. In order to develop and evaluate new therapeutic strategies longitudinal assessment of a tested animal is essential. Non-invasive imaging with PET displays a useful tool for therapy monitoring. However, different mouse models for AD show contradictory findings in ^18^F-FDG-PET.

In this study we review previous use of ^18^F-FDG-PET in different AD mouse models in order to evaluate the impact of PET imaging in preclinical AD research (Tables 1, 2).

**Table 1:** Characteristics of AD mouse models used in ^18^F-FDG-PET studies.

**Mouse line**	**Genetics**	**Key features (months)**	**References**
**APP-BASED MOUSE MODELS OF AD**			
Tg2576	APP mutation: Swedish	•Intraneuronal Abeta (1.5 m) •Inflammation (2 m) •Memory deficits (4 m) •Synaptic dysfunction (4 m) •Plaques (11 m)	(4–6)
**APP AND PSEN DOUBLE TRANSGENIC MICE**			
TASTPM	•APP mutation: Swedish •PSEN1 mutation: M146V	•Plaques (6 m) •Inflammation (6 m) •Memory deficits (6 m)	(7, 8)
APP/PS1	•APP mutation: Swedish •PSEN1 mutation: deltaE9	•Plaques (6 m) •Inflammation (3 m) •Synaptic dysfunction (4 m) •Memory deficits (12 m) •Neuron loss (8 m)	(9–11)
APPPS1-21	•APP mutation: Swedish •PSEN1 mutation: L166P	•Plaques (1.5 m) •Phosphorylated tau, no mature tangles •Neuron loss (17 m) •Inflammation (1.5 m)	(12)
APP/PS2	•APP mutation: Swedish •PSEN2 mutation: N141I	•Plaques (6 m) •Inflammation (6 m) •Synaptic dysfunction (10 m) •Memory deficits (8 m)	(13–15)
5XFAD	•APP mutation: Swedish, Florida, London •PSEN1 mutation: PSEN1, L286V	•Plaques (1.5 m) •Synaptic dysfunction (4 m) •Neuron loss (9 m) •Inflammation (2 m) •Memory deficits (4 m) •Intraneuronal Abeta (1.5 m)	(16, 17)
**APP. PSEN AND TAU TRANSGENIC MICE**			
3xTg	•APP mutation: Swedish •PSEN1 mutation: M146V •Tau mutation: MAPT P301L	•Plaques (6 m) •Tau pathology (12 m) •Intraneuronal Abeta (3 m) •Synaptic dysfunction (6 m) •Memory deficits (4 m) •Inflammation (7 m)	(18, 19)
PLB1 Triple	•APP mutation: Swedish, London •PSEN1 mutation: A246E •Tau mutation: MAPT P301L, R406W	•Plaques (21 m) •Hyperphosphorylated tau (6 m) •Intraneuronal Abeta (12 m) •Synaptic dysfunction (12 m) •Memory deficits (12 m) •Inflammation (12 m)	(20–22)
**NON-APP-BASED MODEL**			
Tg4-42	Overexpressing Aβ4-42 (no mutation)	•Neuron loss (5 m) •Synaptic dysfunction (2 m) •Memory deficits (5 m) •Inflammation (2 m) •Intraneuronal Abeta (2 m)	(23–25)

**Table 2:** Results of ^18^F-FDG-PET studies in AD mouse models.

**Mouse line**	**Age (months)**	**Sex**	**Whole brain FDG-Uptake**	**Regional FDG-Uptake**	**Glucose corrected**	**Normalization**	**References**
**APP-BASED MOUSE MODELS OF AD**							
Tg2576	13–15 m	♂♀	ns	ns	No	Ratio target region/whole brain	(26)
	1) 7 m 2) 19 m	♀♀	1) ↑ 2) ns	1) ↑ 2) ns	No	No	(27)
	18 m	♂♀	ns	↓	Yes	No	(28)
**APP AND PSEN DOUBLE TRANSGENIC MICE**							
TASTPM	14 m	♂	NIA	↓	Yes	No	(29)
	1) 3 m 2) 6 m 3) 9 m 4) 12 m 5) 15 m	♂	NIA	1) ns 2) ns 3) ↓ 4) ns 5) ns	Yes	No	(30)
APP/PS1	1) 2 m 2) 3.5 m 3) 5 m 4) 8 m	♀	NIA	1) ↑ 2) ↑ 3) ↑ 4) ↑	No	Ratio target region/cerebellum	(31)
	1) 3 m 2) 6 m 3) 12 m	♀	NIA	1) ↓ 2) ↑ 3) ↑	Yes	Ratio target region/cerebellum	(32)
APPPS1-21	12 m	♀	NIA	↓	Yes	No	(33)
	12 m		↓	↓	NIA	No	(34)
APP/PS2	1) 5 m 2) 16 m	NIA	NIA	1) ↑ 2) ↑	No	Ratio target region/cerebellum	(35)
5XFAD	11 m	NIA	↑	NIA	No	Ratio target region/cerebellum	(36)
	1) 2 m 2) 5 m 3) 13 m	♂	1) ns 2) ns 3) ↓	1) ns; ↑ 2) ns; ↓ 3) ↓; ↓	No	No; Target region to various regions ratios	(37)
**APP. PSEN AND TAU TRANSGENIC MICE**							
3xTg	1) 6 m 2) 12 m	NIA	1) ↓ 2) ↓	NIA	Yes	No	(38)
PLB1 Triple	1) 5 m	♂♀	1) ns	1) ↓	No	Ratio target region/whole brain	(20)
	2) 17 m		2) ns	2) ↓			
**NON-APP-BASED MODEL**							
Tg4-42	1) 3–4 m 2) 7–8 m	♀	1) ns 2) ↓	1) ↓ 2) ↓	Yes	No	(39)

### APP-Based Mouse Models of AD

The discovery of mutations in the amyloid precursor protein (APP) and presenilin (PSEN) genes in patients with familiar AD led to the generation of a variety of AD mouse models. However, available data on ^18^F-FDG-PET in AD mouse models is scarce.

### ^18^F-FDG-PET in APP Single Transgenic Mice

#### Tg2576

The Tg2576 model is one of the first transgenic mouse model based on mutant APP overexpressing human APP with the double Swedish mutation (K670N/M671L) under the control of the hamster prion promoter (40). Tg2576 mice show a distinct Aβ plaque pathology with increased inflammation by 12 monthsof age. Dendritic spine loss was detected starting at 4.5 months. However, neuron loss is very limited. Noticeably, these mice develop cognitive impairments prior to a significant plaque pathology (41, 42).

Studies with ^18^F-FDG-PET show varying results. The first study using ^18^F-FDG-PET in Tg2576 mice could not show any significant differences of cerebral glucose metabolism compared to WT (26). Animals were scanned on a microPET Focus220 (Siemens Medical Solutions, Knoxville, TN, USA) after injection of ^18^F-FDG to anesthetized mice. Quantification was performed using manual volumes of interest (VOIs) with the help of a 3D digital mouse phantom.

Another study by Luo et al. (27) detected an increase of FDG uptake in 7 months old Tg2576 mice while older mice (19 months) did not show differences compared to WT. In this study ^18^F-FDG was injected to anesthetized mice. Mice were fasted for at least 6 h prior to the study. For dynamic measuring of FDG uptake, images were acquired immediately after injection and continued for 60 min on an Inveon microPET/CT (Siemens Medical Solutions, Knoxville, TN, USA). Quantification was done using whole brain VOIs as well as 7 anatomic regions (cortex, cerebellum, thalamus, hippocampus, striatum, perirhinal cortex and entorhinal cortex) according to a mouse brain atlas. PET results were also compared to cerebral blood volume measured by functional MRI but Tg2576 mice did not show hemodynamic differences compared to age-matched WT mice (27).

In contrast, a more recent study by Coleman et al. (28) detected reduced FDG uptake in 18 months old Tg2576 mice when blood glucose levels were taken into consideration. In this study mice were fasted for 24 h, sampled for blood glucose levels and FDG was injected intraperitoneally while awake. Images were acquired on an Inveon microPET/CT (Siemens Medical Solutions, Knoxville, TN, USA). Results were also confirmed by autoradiography studies of selected animals that were used for *ex vivo* analyses after PET scanning (28). The authors emphasize the importance of blood glucose corrections of SUV as blood glucose levels can affect brain FDG uptake and transgenic mice are known for a significantly greater decline of blood glucose levels after fasting compared to WT (29).

### ^18^F-FDG-PET in APP/PSEN Double Transgenic Mice

In order to create a more prominent AD pathology mouse models with APP and PSEN or a combination of multiple mutations were generated. Generally speaking, double transgenic mice expressing mutated PSEN and APP develop an earlier and more aggressive pathology with severe plaque pathology, behavior impairments and increased inflammation (41, 43, 44).

#### APPPS1-21

APPPS1-21 mice co-express the human Swedish mutation KM670/671NL and PSEN1 L166P under the control of the neuron-specific Thy-1 promoter (44). Mice show age-dependent Aβ plaque depositions in the hippocampus starting as early as 6 weeks of age accompanied by increased astrogliosis and microgliosis. Dendritic spine loss occurs around plaques beginning 4 weeks after plaque formation, continuing for several months. Modest neuron loss could be detected in the dentate gyrus in 17 months old mice. Cognitive impairment is reported at 7 months (45).

Two studies used ^18^F-FDG-PET in APPPS1-21 mice so far (33, 34). Twelve months old APPPS1-21 mice showed significantly reduced FDG uptake in the thalamus and striatum. Anesthetized animals were scanned after a conscious uptake period of 45 min on a microPET/CT (Siemens Medical Solutions, Knoxville, TN, USA). Animals were fasted overnight for 8–12 h and blood glucose was monitored. Quantification was performed in predefined VOIs with the help of a mouse brain MRI template and glucose-corrected uptake values were calculated. *Ex vivo* autoradiography was performed confirming *in vivo* findings. Furthermore, FDG uptake was compared to amyloid burden but did not show a correlation (33). The second study of Takkinen et al. (34) confirmed those findings. Twelve months old APPPS1-21 mice showed significantly decreased FDG uptake in the whole brain and in several regions including cortex, hippocampus, striatum, thalamus and cerebellum. Mice were fasted for 90 min and anesthetized 30 min prior tracer injection. Dynamic images were acquired on an Inveon PET/CT scanner (Siemens Medical Solutions, Knoxville, TN, USA) for 60 min. VOIs were defined with the help of an MRI mouse brain template and SUVs were calculated. Furthermore, FDG uptake was compared to imaging of neuroinflammation using the tracer ^18^F-DPA-714. FDG uptake correlated positively with ^18^F-DPA-714 in the cortex and hippocampus of 6 months old mice (34).

#### APP/PS1

The APP/PS1 model co-expresses the Swedish mutation KM670/671NL and PSEN1 delta E9. Mice show age-dependent extracellular Aβ deposition starting at 6 months of age. Mice show age-dependent cognitive impairment starting at 12 months.

First signs of astrocytosis can be detected at 3 months of age. However, severe gliosis starts around 6 months, especially in close proximity to plaques (9–11, 46).

So far two studies scanned APP/PS1 mice with ^18^F-FDG-PET showing an age-dependent increase of glucose metabolism. Poisnel et al. (32) detected a significantly higher FDG uptake in the cortex and hippocampus of 12 months old animals compared to WT scanning 3, 6, and 12 months old APP/PS1 mice. Mice were scanned on a microPET Focus 220 (Siemens Medical Solutions, Knoxville, TN, USA). FDG was injected to anesthetized mice followed by a 60 min uptake period maintaining anesthesia. Blood glucose was measured and levels were in normal range. Four VOIs were defined (cortex, hippocampus, striatum and cerebellum) with the help of representative MRI scans and SUV values were calculated in each VOI. SUVs were normalized by cerebellum uptake. Furthermore, functional MRI showed cortical hypoperfusion in 12 months old APP/PS1 mice (32).

Another study by Li et al. (31) showed increased FDG uptake in several brain regions in 2, 3.5, 5, and 8 months old APP/PS1 mice. Mice were scanned on an Inveon microPET/CT (Siemens Medical Solutions, Knoxville, TN, USA). Mice were fasted at least 6 h prior to scanning. During injection and the uptake period, mice were awake. VOIs were defined manually on MRI slices of each animal and SUV values were calculated in each VOI and were normalized by cerebellum uptake (31).

#### PS2APP

The PS2APP model co-expresses human presenilin 2 (N141I mutation) and human APP (K670N/M671L mutation). PS2APP mice display age-dependent Aβ plaque pathology accompanied by gliosis starting at 6 months of age. Cognitive impairment was detected starting at 8 months (13–15).

A triple tracer study using ^18^F-FDG, the amyloid tracer ^18^F-Florbetaben and the neuroinflammation tracer ^18^F-GE180 showed increased FDG uptake in 5 and 16 months old PS2APP mice. Images were acquired on an Inveon microPET/CT (Siemens Medical Solutions, Knoxville, TN, USA). VOIs were defined using an MRI mouse brain atlas and SUV ratios using the cerebellum for reference were calculated. SUVr (forebrain/cerebellum) was higher in PS2APP mice compared to WT. Furthermore, SUVr correlated with microgliosis and amyloid load while microgliosis and amyloid load strongly correlated (35).

#### TASTPM

The double transgenic model TASTPM carries the Swedish mutation in APP and the M146V mutation in PSEN1. While sporadic Aβ deposits can be detected in 3 months old TASTPM mice, extracellular Aβ plaque depositions are not evident before 6 months of age. Astrogliosis and microgliosis around amyloid plaques could be detected by 6 months of age. Age-dependent cognitive impairment is described starting at 6 months (7, 8).

Three studies using ^18^F-FDG-PET in TASTPM mice performed by the same group showed significant regional decreased FDG uptake in 9 and 14 months old animals compared to WT. ^18^F-FDG-PET/CT was performed on an Inveon microPET/CT (Siemens Medical Solutions, Knoxville, TN, USA). Mice were fastened for 8–12 h before PET. ^18^F-FDG was injected to awake mice and images were acquired after a conscious uptake period of 45 min. Blood glucose levels were measured and used for glucose normalization of SUV. Uptake values were measured in predefined VOIs using a mouse MRI atlas. Furthermore, these studies emphasized the blood glucose normalization in order to compensate effects on FDG uptake and differences in fasting durations. However, decrease of FDG uptake did not progress over age and did not correlate with Aβ plaque pathology (29, 30, 47).

#### 5XFAD

The widely used 5XFAD model co-expresses five mutations of familial AD overexpressing human APP and PSEN-1. 5XFAD mice develop plaque pathology accompanied by astrocytosis and microgliosis starting at 2 months of age with massive increase over age. Starting at 9 months of age, 5XFAD mice exhibit significant synaptic loss. Neuron loss is detectable in cortical layer 5 and subiculum. Mice show cognitive impairment starting at 4 months (16, 17).

Studies with ^18^F-FDG-PET show varying results. Rojas et al. (36) detected an increased uptake of ^18^F-FDG in 11 months old 5XFAD mice compared to WT. In this study ^18^F-FDG was injected while mice were anesthetized with isoflurane and images were acquired after an awake uptake period of 50 min on a Concorde Microsystems microPET (Concorde Microsystems, TN, USA). Mice were not fasted in advance to PET. Manually defined VOIs were used to calculate whole brain to cerebellum uptake ratios. The authors assumed that those findings might be explained by reactive changes of microglia and astrocytes according to inflammatory processes in the brain due to excessive brain amyloidosis (36).

Another study by Macdonald et al. (37) reported a significant decrease of ^18^F-FDG uptake in 13 months old 5XFAD mice. Younger mice tested at 2 and 5 months did not show significant differences in whole brain SUV compared to WT. In this study ^18^F-FDG was injected to conscious mice. During the uptake period mice interacted with a mechanical mouse. Images were acquired after an uptake period of 30 min on a LabPET4 preclinical PET/CT scanner (Trifoil Imaging, CA, USA). SUV values were measured in 8 predefined VOIs (amygdala, basal forebrain, basal ganglia, cerebellum, hippocampus, hypothalamus, neocortex, and thalamus) with the help of an MRI mouse brain atlas and standard uptake values (SUVs) were calculated. Thirteen months old 5XFAD mice showed significantly lower SUV in all brain regions. Furthermore, relative metabolic activity was calculated by using ratios between different brain regions. Young 5XFAD mice showed differences to WT in the basal forebrain, hippocampus, hypothalamus and thalamus relative to the neocortex as a possible early indicator of regional changes (37). Unpublished data of our own group also show significant decreases of ^18^F-FDG uptake in 12 months old 5XFAD mice.

### ^18^F-FDG-PET in Other Mouse Models of AD (APP + PSEN + tau)

While APP and PSEN mouse models mirror a wide spectrum of the pathological features seen in AD patients they lack a robust neurofibrillary tangle (NFT) pathology. In order to overcome this absence familial AD mutations have been combined with tau mutations from frontotemporal dementia.

#### 3xTg

The 3xTg model is an example for such a triple transgenic mouse model as these mice show NFT pathology together with plaque depositions. 3xTg mice express three genes associated with familial AD namely human APP with the Swedish mutation, PSEN1 M146V together with mutated Tau P301L (18).

One study used ^18^F-FDG-PET in 3xTg mice. Sancheti et al. (38) detected significantly decreased FDG uptake in 6 months old 3xTg mice compared to WT. Mice were fasted overnight and anesthetized before PET. Images were acquired on a microPET R4 PET scanner Siemens Medical Solutions, Knoxville, TN, USA) and additional CT scans were performed. VOIs were drawn manually in co-registered PET and CT images and SUVs were calculated. In addition, the effect of lipoic acid administration on glucose uptake was evaluated and showed an increase of FDG uptake in lipoic acid fed 3xTg mice (38).

#### PLB1

The PLB1_Triple_ line is also a triple transgenic AD mouse line carrying human mutated APP with the London and Swedish mutation, mutated tau (P301L/R406W) as well as mutant PSEN1. PLB1_Triple_ mice demonstrate a slow but progressive AD-like pathology. While intraneuronal Aβ can be detected in 12 months old mice Aβ plaques are not visible before 21 months of age. First memory deficits have been observed in 12 months old PLB1_Triple_ mice (20).

Platt et al. (20) detected decreased bilateral FDG uptake in the occipital and parietal cortex of 5 and 17 months old PLB1 mice extending to the prefrontal cortex and cerebellum at 17 months. Mice were fasted overnight prior scanning.^18^F-FDG was injected intraperitoneally to conscious mice. Scans were performed on a Suinsa ARGUS dual-ring scanner (Suinsa Medical Systems, Madrid, Spain). Voxel-based analysis was performed using a digital mouse brain atlas with normalization to whole brain uptake. Regions of statistical significance were mapped on CT images (20).

### ^18^F-FDG-PET in Non-APP-Based Models of AD

Familial AD accounts only for a small fraction of all AD cases, however all of the above described AD mouse models rely on one or more FAD mutations. Most AD patients suffer from the late-onset sporadic form of the disease (LOAD) that is not linked to mutations. Therefore, development of animal models that reflect LOAD are becoming into focus.

#### Tg4-42

The transgenic mouse model Tg4–42 is unique as it overexpresses Aβ4-42 without harboring mutations in the Aβ sequence. Tg4-42 mice express human Aβ4-42 fused to the murine thyrotropin-releasing hormone signal peptide, ensuring secretion through the secretory pathway, under the control of the neuronal Thy-1 promoter. Tg4-42 mice show intracellular soluble neurotoxic aggregates of Aβ4-42, without the formation of extracellular amyloid plaques. Homozygous mice show significant age-dependent neuron loss in the hippocampus starting at 5 months with associated astrogliosis and microgliosis at 2 months. Mice show age-dependent cognitive impairment starting at 5 months (23, 24).

**Figure 1:**
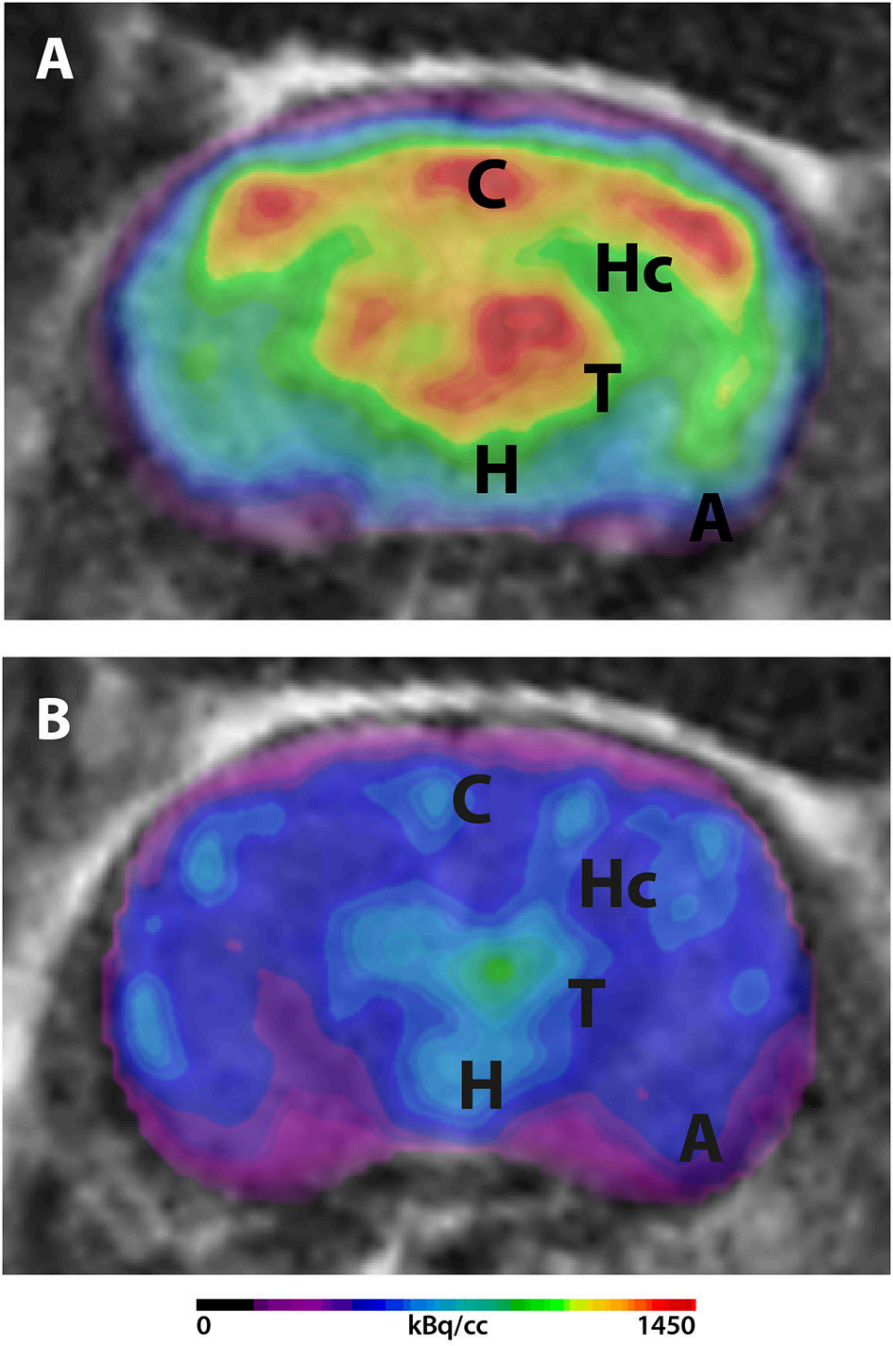
^18^F-FDG-PET in Tg4-42 mice. Mice were fasted overnight and ^18^F-FDG (mean 16.2 MBq) was administered intravenously. Scans were performed after an awake uptake period of 45 min. Mice were anesthetized with isoflurane for the injection and during the scans. PET images were acquired on a small animal 1 Tesla nano scan PET/MRI (Mediso, Hungary) for 20 min. A 136 × 131 × 315 matrix with a voxel size of 0.23 × 0.3 × 0.3 mm^3^ was used. MRI images were used for attenuation correction (matrix 144 × 144 × 163 with a voxel size of 0.5 × 0.5 × 0.6 mm^3^, TR: 15 ms, TE 2.032 ms and a flip angle of 25°). Fused PET/MRI images are shown in coronal view. **(A)** ^18^F-FDG-PET/MRI of a wildtype mouse with homogenous FDG-distribution in all brain areas. **(B)** ^18^F-FDG-PET/MRI of an aged Tg4-42 mouse with distinct lower uptake compared to wildtype mice. A, amygdala; C, cortex; H, hypothalamus; Hc, hippocampus; T, thalamus.

A recent study of our group detected age-dependent reduction of ^18^F-FDG uptake in Tg4-42 mice. Mice were scanned as described above. Early changes in FDG uptake were detected in the hippocampus, forebrain, hypothalamus, amygdala and midbrain at 3 months of age progressing to all brain areas at 8 months. Old Tg4-42 mice showed differences in SUVGlcin all tested brain regions and whole brain compared to WT (39). Mice were scanned on a small animal PET/MRI scanner (1 Tesla nanoScan PET/MRI; Mediso, Hungary). Mice were fasted overnight and blood glucose levels were measured prior to tracer injection. ^18^F-FDG was injected while mice were anesthetized with isoflurane and images were acquired after an awake uptake period of 45 min. A mouse brain atlas template was used to analyze 11 different predefined brain regions (amygdala, brain stem, cerebellum, cortex, hippocampus, hypothalamus, midbrain, olfactory Bulb, septum/Basal Forebrain, striatum, and thalamus). SUV was calculated and values were corrected for blood glucose levels in order to stabilize uptake values compensating for inequalities in glucose levels and fasting durations (Figure 1).

## Discussion

Animal models of AD are essential in order to understand underlying pathologies of the disease and develop new therapeutic strategies. In order to study new therapies, longitudinal assessment of their effects *in vivo* is important and depends on disease-specific biomarkers. ^18^F-FDG-PET is an established imaging biomarker for neuronal dysfunction in the diagnostic workup of AD-patients. ^18^F-FDG-PET displays cerebral glucose metabolism which is mainly determined by synaptic activity. Changes in glucose metabolism can be detected in early stages of the disease as neuronal dysfunction precedes neuron loss. Successful translation of preclinical findings into clinical use requires animal models that display key features of the disease and allow the use of common biomarkers.

Multiple studies using ^18^F-FDG-PET in animal models of AD have been carried out so far and results of those studies are very inconsistent questioning whether ^18^F-FDG-PET is a suitable method in AD mouse models. Here we want to summarize earlier findings and discuss possible explanations of those results and their discrepancies.

In contrast to typical findings in AD patients, several studies described increased FDG uptake in transgenic AD mice including the 5XFAD, Tg2576, APPS1-21, and APP/PS1 models (27, 32, 35, 36). One study detected unaltered glucose metabolism in Tg2576 mice (26) while other studies showed decreased cerebral FDG uptake in 5XFAD, Tg2576, APPS1-21, TASTPM, Tg4-42, PLB1, and 3xTg mice (28, 30, 33, 34, 37–39).

The mainly used explanation of increased FDG uptake is the presence of inflammatory cells around amyloid plaques. High astrogliosis and microgliosis might lead to an increased glucose metabolism. However, contradicting results in the same animal models (data available for 5XFAD and Tg2576 mice) as well as in other models with known increased gliosis (APPS1-21, TASTPM, and Tg4-42 mice) showed significant cerebral hypometabolism relativizing this theory. Furthermore, AD patients also display distinct decreased brain glucose metabolism despite highly increased gliosis (48–50). Therefore, astrogliosis and microgliosis alone do not seem to be responsible for cerebral hypermetabolism.

Other explanations for high FDG uptake in the brain include vascular alterations and changes of the blood brain barrier. Next to FDG-PET, Poisnel et al. (32) used functional MRI to measure cerebral perfusion in APP/PS1 mice that showed increased FDG uptake. However, they detected an even reduced cortical perfusion showing that perfusion changes are not accountable for lower FDG uptake in this model (32). Changes in the blood brain barrier as described in histology for Tg2576 mice might lead to increased leakage of FDG into the brain (51). However, two studies on Tg2576 mice did not show a relevant effect of FDG leakage as FDG uptake was decreased or comparable to WT.

Methodological differences should also be taken into consideration. The most striking methodological differences between the studies are found in normalization methods of PET results. Normalization to the cerebellum is a common method as disease pathologies are expected to mainly influence cortical regions. Cerebellar glucose metabolism is relatively preserved in AD patients and normalization with the cerebellum could be shown to improve differential diagnosis of dementia (52–54). However, in mice several studies also describe lower FDG uptake in the cerebellum and therefore it seems to be somehow affected by the pathologies as well (29, 30, 39). Deleye et al. (29) described that normalization to the cerebellum yielded in higher SUVs in transgenic mice. Therefore, the cerebellum seems not appropriate as a reference region for FDG-PET results. Interestingly, all studies that normalized SUVs with the cerebellum did show increased FDG uptake compared to WT independent of the used mouse model. Unfortunately, data of non-normalized SUVs were not shown.

Another common method of normalization includes blood glucose levels. FDG uptake in the brain is highly influenced by blood glucose levels showing an inverse relationship with FDG-uptake (55). Several factors as fasting and fasting duration, stress, body temperature and anesthesia with isoflurane or ketamine influence blood glucose levels. Next to providing stable pre-imaging conditions, normalization of FDG uptake for blood glucose levels should be taken into consideration as some factors cannot always be perfectly stabilized in each individual animal. Furthermore, transgenic mice show lower blood glucose after fasting compared to WT mice (28, 29). This effect might mimic higher FDG uptake in the brain compared to WT.

Further methodological differences between studies include animal preparation and handling (e.g., fasting times), conditions during the uptake period and anesthesia. These differences can influence FDG uptake due to changes in blood glucose levels as mentioned above and some studies used glucose normalization to exactly overcome these issues. Furthermore, synaptic activity might differ between studies due to the use of anesthesia or stimulation (e.g., room conditions, mechanical companion mouse). However, these conditions should not affect results as conditions have to be stable within a study for both, WT and transgenic mice.

Furthermore, technical aspects also differ between studies including PET scanners, acquisition times and image analysis. Spatial resolution displays a major challenge for small animal imaging, especially in the brain. While autoradiography can provide a spatial resolution around 100 μm and below common modern PET scanners and reconstruction engines reach a spatial resolution of around 1.5 mm up to 700 μm in the newest systems (56). While regional differences between autoradiography and small animal PET studies can be explained by the inferiority of PET scanners in terms of spatial resolution, differences between PET scanners in the reviewed studies are not that critical and therefore varying results cannot be majorly explained by the use of different systems.

Pathological features of different mouse models might also influence FDG-PET results. Lacking cerebral hypometabolism may also be due to a limited amount of neuron loss. While atrophy and neuron loss as major hallmarks of AD mainly influence FDG uptake in humans, some transgenic APP-based animal models lack this key feature. However, FDG uptake is not only determined by neuron loss. Synaptic dysfunction also leads to lower glucose consumption in the brain. Studies on mouse models that only show minimal or even lack neuron loss as Tg2576, APPPS1-21, or TASTPM do show cerebral hypometabolism indicating that neuron loss is not the only factor driving FDG uptake.

To date, the impact of ^18^F-FDG-PET in preclinical studies is subordinate. However, in future studies small animal imaging with ^18^F-FDG-PET should become an important biomarker for therapy monitoring. In studies on new therapeutic approaches a suitable mouse model that can be monitored by ^18^F-FDG-PET can be beneficial, especially for monitoring early therapeutic success.

## Conclusion

^18^F-FDG-PET can be a valuable tool for longitudinal *in vivo* monitoring of AD mice considering its strengths and limitations. Suitability of a certain animal model regarding its pathological features as well as normalization techniques should be carefully considered. Normalization with blood glucose in order to compensate for fasting and preparation inequalities that highly influence blood glucose levels in transgenic mice seems helpful whereas normalization with the cerebellum seems inappropriate.

## Author Contributions

CB and YB wrote the manuscript. All authors approved the final version.

### Conflict of Interest Statement

The authors declare that the research was conducted in the absence of any commercial or financial relationships that could be construed as a potential conflict of interest.
